# The importance of sleep quality, quantity, and chronotype in the management of diabetes: Is it time to wake up?

**DOI:** 10.1111/1753-0407.13313

**Published:** 2022-09-09

**Authors:** Theocharis Koufakis, Giuseppe Maltese, Djordje S. Popovic, Kalliopi Kotsa

**Affiliations:** ^1^ Division of Endocrinology and Metabolism and Diabetes Center, First Department of Internal Medicine, Medical School Aristotle University of Thessaloniki, AHEPA University Hospital Thessaloniki Greece; ^2^ Department of Diabetes and Endocrinology Epsom & St Helier University Hospitals Surrey UK; ^3^ Unit for Metabolic Medicine, Cardiovascular Division, Faculty of Life Sciences & Medicine King's College London UK; ^4^ Clinic for Endocrinology, Diabetes and Metabolic Disorders, Clinical Centre of Vojvodina, Novi Sad, Serbia; Medical Faculty University of Novi Sad Novi Sad Serbia

To the editors,

We read with great interest the synopsis of the 2022 meeting of the American Diabetes Association by the Editor‐in‐Chief of the *Journal of Diabetes*, Professor Z. Bloomgarden.^1^ In addition to exciting and novel research data, a highlight of the congress was the presentation of the draft of the new American Diabetes Association/European Association for the Study of Diabetes (EASD) consensus on the management of hyperglycemia in type 2 diabetes (T2D).^2^ The consensus is expected to be shaped in its final form at the upcoming EASD congress, which will be held in Stockholm, Sweden, in September 2022.

Undoubtedly, we live in the era of the best pharmacological treatments we have ever had for the management of T2D. Sodium‐glucose cotransporter 2 inhibitors and glucagon‐like peptide 1 receptor agonists can effectively lower blood glucose with a minimal risk of hypoglycemia, promote weight loss, and reduce the cardiorenal risk. And with tirzepatide already available in clinical practice, exciting times are likely to come. However, lifestyle modifications continue to represent a key pillar of diabetes management. For many years, healthcare professionals in diabetes care have focused their attention exclusively on diet and physical activity. The new consensus adequately raises the importance of paying attention to 24‐h physical behaviors of people with diabetes and incorporates recommendations on quality and quantity of sleep, as well as chronotype.

Emerging evidence suggests that sleep parameters can significantly affect the glycemic status. A meta‐analysis of 19 observational studies showed that both long and short sleep duration could have an adverse impact on diabetes control, with the former associated with higher fasting plasma glucose (FPG) and the latter with higher levels of glycated hemoglobin.^3^ Furthermore, good compared to poor sleep quality was correlated with significantly lower FPG concentrations. Chronotype is defined as the natural inclination of an individual with respect to the times of day when they prefer to sleep. Hashemipour et al. have recently shown that the evening chronotype (characterized by insomnia and short sleep duration) is associated with poorer glycemic control.^4^ The relationship between sleep disturbances and diabetes decompensation is certainly complex and has been only partially elucidated: overactivation of the sympathetic system, upregulation of oxidative stress, changes in levels of appetite regulation hormones, and unhealthy eating habits are a few of the underlying putative mechanisms.^5^ Obstructive sleep apnea is also strongly linked to diabetes, although it is dubious which one develops first, given the bidirectional relationship between the two entities.^6^

To summarize, the new consensus on the management of hyperglycemia in T2D encourages a holistic approach to patient care. Regarding specifically the lifestyle change that is necessary for people with diabetes, this goes beyond the traditional (but very insufficient considering the available evidence) tactic of “eat less, move more.” Evaluation of sleep quality and other relevant parameters should gradually be incorporated into our daily clinical routine, and future studies should focus on sleep interventions that can improve both glycemic control and quality of life for people with T2D. In other words, it is time for the diabetes specialist community to wake up!

## AUTHOR CONTRIBUTIONS

Theocharis Koufakis has received honoraria for lectures from AstraZeneca, Boehringer Ingelheim, Pharmaserve Lilly, and Novo Nordisk; for advisory boards from Novo Nordisk; and has participated in sponsored studies by Eli‐Lilly and Novo Nordisk. Giuseppe Maltese has received honoraria for lectures from AstraZeneca and Novo Nordisk. Djordje S. Popovic declares associations to Abbott, Alkaloid, AstraZeneca, Boehringer‐Ingelheim, Berlin‐Chemie, Eli Lilly, Galenika, Krka, Merck, Novo Nordisk, PharmaSwiss, Sanofi‐Aventis, Servier, Viatris, and Worwag Pharma. Kalliopi Kotsa has received honoraria for lectures/advisory boards and research support from Astra Zeneca, Boehringer Ingelheim, Pharmaserve Lilly, Sanofi‐Aventis, ELPEN, MSD, and Novo Nordisk.
